# Presence of interictal epileptiform EEG discharges implies increased risk of recurrence after the first unprovoked seizure: Report of the International League Against Epilepsy and International Federation of Clinical Neurophysiology

**DOI:** 10.1111/epi.18591

**Published:** 2025-08-28

**Authors:** Betül Baykan, John Dunne, Samuel Wiebe, Louis Maillard, Sandor Beniczky, Michalis Koutroumanidis, Margitta Seeck

**Affiliations:** ^1^ Department of Neurology, Istanbul Faculty of Medicine Istanbul University Istanbul Turkey; ^2^ EMAR Medical Centre Istanbul Turkey; ^3^ Neurology Department Royal Perth Hospital Perth Western Australia Australia; ^4^ Discipline of Internal Medicine, Medical School The University of Western Australia Perth Western Australia Australia; ^5^ Department of Clinical Neurosciences University of Calgary Calgary Alberta Canada; ^6^ CNRS, University Hospital of Nancy University of Lorraine Nancy France; ^7^ Danish Epilepsy Centre Dianalund Denmark; ^8^ Aarhus University Aarhus Denmark; ^9^ Department of Neurology and Clinical Neurophysiology, St. Thomas' Hospital Kings College London London UK; ^10^ EEG & Epilepsy Unit, Department of Clinical Neurosciences University Hospital of Geneva Geneva Switzerland

**Keywords:** EEG, first seizure, new‐onset epilepsy, relapse, risk

## Abstract

**Objective:**

A joint International Federation of Clinical Neurophysiology–International League Against Epilepsy (IFCN‐ILAE) Taskforce was created to explore the published evidence for initial EEG recordings in the evaluation of patients who experienced their first unprovoked seizure, and to determine the diagnostic value of EEG in supporting the diagnosis of epilepsy.

**Methods:**

We conducted a systematic literature review, with two independent authors screening each study. We extracted seizure recurrence data among patients with EEG showing interictal epileptiform discharges (IEDs) vs those with normal or nonspecific‐abnormal EEG results. Random‐effects meta‐analyses of seizure recurrence in relation to IEDs was conducted in the included studies, calculating odds ratios (OR) with confidence intervals (CIs) and diagnostic accuracy.

**Results:**

A total of 4847 patients from 22 studies with variable follow‐up durations were analyzed. The random‐effects pooled binary estimate of seizure recurrence was 47% (95% CI 40%–55%). The overall proportion with seizure recurrence was higher in patients with IEDs (60%, 95% CI 53%–68%) compared to those without (40%, 95% CI 33%–48%, *p* < .001). Random‐effects meta‐analysis showed that the presence of IEDs was associated with seizure recurrence (OR 2.32, 95% CI 1.69–3.17, *p* < .001). Subgroup analyses of adults and children showed that this difference remained significant in both groups: OR in children of 3.24 (95% CI 2.19–4.79) and in adults of 1.55 (95% CI 1.08–2.21). In eight studies (*n* = 1209, 923 children) patients remained untreated before the second seizure; the pooled probability of seizure recurrence in those with IED in these studies was no different than in studies in which some patients were treated.

**Significance:**

In conclusion, the presence of IEDs in EEG recordings obtained after the first unprovoked seizure can help clinicians to confirm the clinical diagnosis of epilepsy after a first unprovoked seizure, according to the revised ILAE definition. These results support the relevance of IED detection on EEG as a predictor of seizure recurrence after a first unprovoked seizure. However, its prognostic value is influenced by age and other clinical factors.

**Key points**

- If the initial EEG after a first unprovoked seizure shows interictal epileptiform discharges (IEDs), the risk of seizure recurrence increases in both children and adults.
- The presence of IEDs in EEG recordings may exceed the current threshold of 60% probability of recurrence recommended by the International League Against Epilepsy (ILAE) for the diagnosis of epilepsy, aiding clinicians in confirming the diagnosis of epilepsy after a first unprovoked seizure.
- Several areas still require further investigation, including the optimal timing of EEG after the event, the duration of EEG recording, activation strategies (such as awake vs sleep EEG), and subgroup analyses, including accounting for the impact of antiseizure medications.

## 1 INTRODUCTION

EEG is a helpful tool in diagnosing epilepsy, supporting what is primarily a clinical diagnosis.^1^ Previously, the diagnosis of epilepsy required at least two unprovoked seizure episodes. However, as per the current operational diagnosis of epilepsy outlined in the position paper of the International League against Epilepsy (ILAE), clinicians can diagnose epilepsy after the first unprovoked seizure if paraclinical data indicate an enduring predisposition to unprovoked seizures with a risk comparable to those having had two unprovoked seizures, using a level of 60% over 10 years.^2^, ^3^ Since publication of the ILAE position paper, data‐driven estimates have shown the 10‐year risk of recurrence in patients having had two unprovoked seizures is 85%,^4^, ^5^, ^6^ leading to some confusion and debate within the medical community. The ILAE position paper mentioned only one example concerning post‐stroke epilepsy, and no further guidance was provided regarding the specific “paraclinical” data to be considered.^2^

Given the importance and extensive use of EEG as a diagnostic tool in patients with epilepsy, we aimed to review the current evidence supporting the role of the initial EEG in the diagnostic evaluation of the first seizure. This study explores the diagnostic added value of EEG in patients experiencing their first unprovoked seizure based on the existing literature to assist clinicians in their decision‐making.

## 2 METHODS

In 2018, a task force comprising seven senior epileptologists and clinical neurophysiologists was assembled by the ILAE and the International Federation of Clinical Neurophysiology (IFCN). This joint IFCN‐ILAE Taskforce aimed to define a protocol to assess the role of routine EEG in the diagnostic evaluation of the first unprovoked seizure.

### 2.1 Literature search, review and eligibility criteria

**FIGURE 1:**
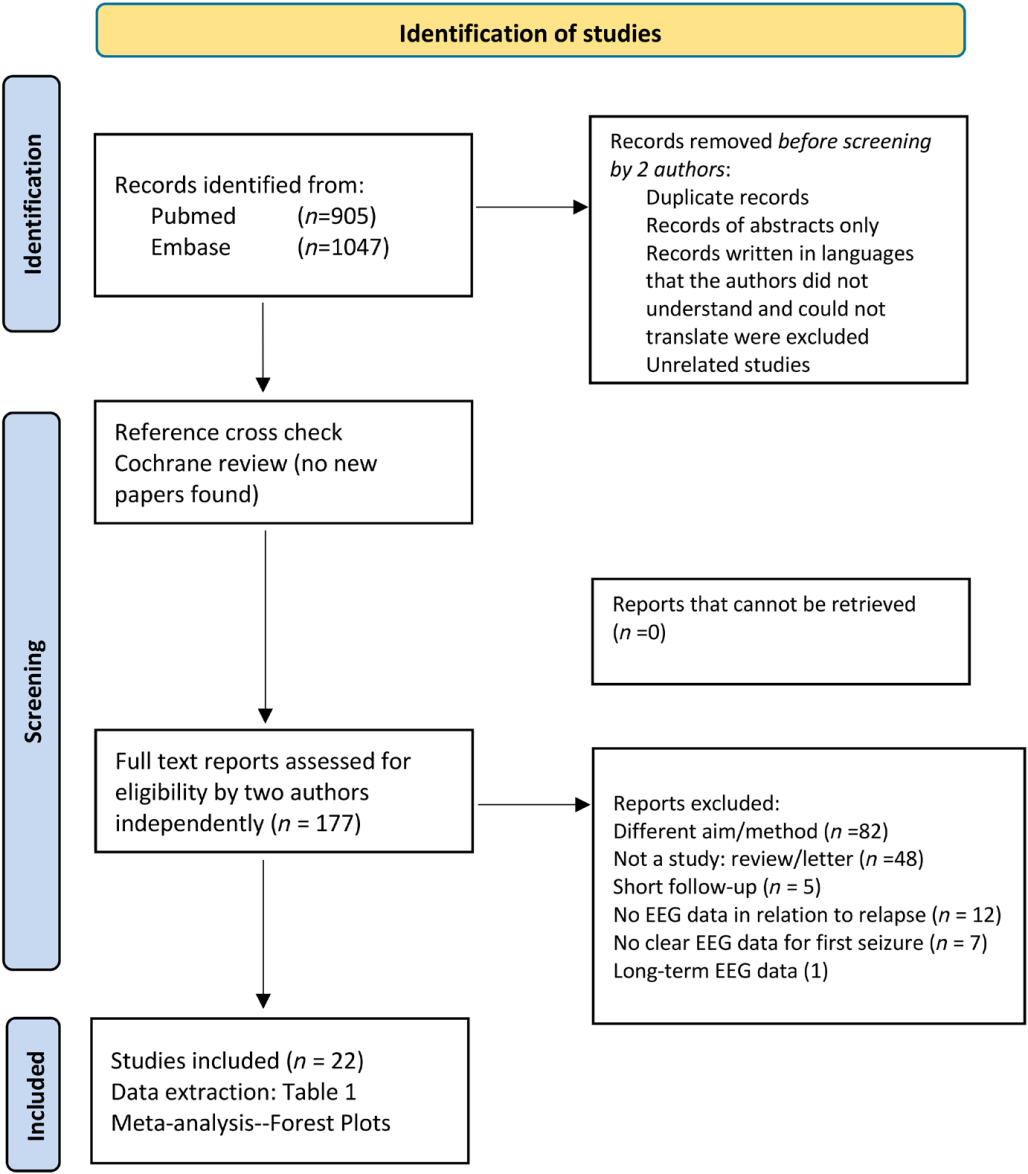
PRISMA flowchart diagram of the study (Preferred Reporting Items for Systematic reviews and Meta‐Analyses).

We used a systematic literature review protocol, outlining the research questions and study inclusion criteria. This systematic review was performed using Preferred Reporting Items for Systematic reviews and Meta‐Analyses (PRISMA) guidelines (Figure 1).

PubMed, Cochrane, and Embase databases were searched for articles reporting on the diagnostic value of EEG in the context of a first seizure, focusing on epileptiform abnormalities. The search employed specific keywords including “EEG,” “electroencephalography,” “first seizure,” and “early onset epilepsy,” along with subject headings (i.e., MeSH, EmTree). We limited the search to human studies and considered publications from 1985 until 2025, with studies requiring for inclusion the use of the 10–20 electrode placement system. Studies were excluded if they lacked sufficient information to fully assess their eligibility (e.g., full text not available or not available in languages that the authors could understand or translate). Letters, commentaries, conference abstracts, poster presentations, and supplementary materials only were excluded. Studies focusing on other topics, such as epilepsy surgery or EEG in the intensive care unit (ICU), were also excluded. Finally, studies were excluded if the sample size was <10, if follow‐up duration was <12 months, or if there was no information on seizure recurrence.

Following the search, the review process was performed independently by two reviewers for each of the papers and discrepancies were resolved through consensus. We removed duplicate publications reporting on the same cohort. This process led to the identification of 177 studies, then subjected to abstract screening for eligibility assessment based on the predefined inclusion and exclusion criteria. Subsequently, a full‐text review of these articles was performed, leading to the inclusion of 22 studies (Table 1).

**TABLE 1:** Studies providing data on the importance of interictal epileptiform discharges (IEDs) in patients with a first unprovoked seizure.

Author, year	Design, target age group	Patient selection criteria	Number of patients analyzed	Age range (mean or median) months/years	Follow‐up duration: number with seizure recurrence	Number remaining seizure free	EEG with IEDs *N* (%)	Number with IEDs of those with seizure recurrence	Number with IEDs of those remaining seizure free	Treated after first seizure (%)	Delay after first seizure until first EEG
Camfield^a^ (1985)^7^	Retrospective, pediatric	EEG lab referrals only Excluded abs, akinetic, myo sz, and infantile spasm, brain tumor or progressive neurological disease	168	1 month–16 years	Mean 2.6 years ± 1.5 years: 87	81	73 (43.5)	48/87	25/81	115 (68%)	NI
Shinnar^a^ (1994)^8^	Prospective, pediatric/adolescent	Excluded abs and myo Sz	321	1 month–19 years (mean: 6.8 years)	Mean 3.9 years: 131	190	102 (31.8)	60/131	42/190	“Most subjects were not treated”	>90% over 48 h after first seizure
Bora^a^ (1995)^9^	Prospective, adults	Only idiopathic GTCS; Excluded focal sz and CT lesions	147	16–66 years (mean: 23.8 years)	2 years: 65	82	51 (34.7)	18/65	33/82	62 (42%)	“Usually over 48 h”
Stroink (1998)^10^	Prospective, pediatric		156	1 month–16 years (mean: 7.1 years; median: 6.9 years)	2 years: 84	72	68 (43.6)	48/84	20/72	No ASM before recurrence	NI
Bessisso (2001)^11^	Prospective, pediatric		33	2 months–12 years (mean: 4 years)	1 year: 11	22	11 (33.3)	7/11	4/22	5 (15%)	NI
Hui (2001)^12^	Retrospective, adults	EEG lab referrals only Only GTCS; excluded focal sz, abs, myo sz, SE, brain lesions	129	13–86 years (mean: 33 years)	Mean 2.3 years: 60	69	39 (30.2)	20/60	19/69	No ASM before recurrence	Mean 15.7 days (range 1–36 days)
Schreiner (2003)^13^	Prospective, adults	Excluded abs, myo, brain tumor, AIDS	157	17–84 years (mean: 48 years)	Mean: 2.8 years (range 3 months–7.3 years): 49	108	42 (26.8)	18/49	24/108	“A negligible number” treated	<2 days
Inaloo (2008)^14^	Prospective, pediatric	Only GTCS; Excluded focal sz, abs, myo sz	142 (with EEG)	2 months–16 years (mean: 6.9 ± 4.4 years; median: 7 years)	Mean (1.2 years ± 4.5 months): 69	73	43 (30.3)	38/69	5/73	96 (67.9%)	NI “as soon as feasible”
Arthur^a^ (2008)^15^	Prospective, pediatric	Neurologically normal children Excluded abs, myo sz	150	6 years–14 years (mean: 9.7 years)	2.25 years: 99	51	85 (56.7)	59/99	26/51	96 (64%)	NI
Pereira^a^ (2014)^16^	Prospective, pediatric	Excluded neonatal sz, epileptic encephalopathy, abs, myo Sz and infantile spasms	200	4 months–10 years (mean: 4.5 years)	2 years: 56	144	82 (41.0)	37/56	45/144	No ASM before recurrence	Within first 3 months
Lawn^a^ (2015)^6^	Prospective, adults		752 (with >1 year follow‐up)	14–91 years (median: 39 years)	Mean 6.1 years (1–15.3 years): 254	498	129 (17.2)	54/254	75/498	212 (27%)	Mean 24 days (median 10 days)
Kanemura (2015)^17^	Prospective, pediatric	Excluded SE (required at least one EEG showing awake‐drowsy‐sleep‐arousal‐awake states. All fully sleep deprived before EEG)	87	3 months–13.4 years (mean: 3.4 years)	4 years: 48	39	52 (59.8)	35/48	17/39	No ASM before recurrence	7–20 days after the first sz
Kim (2016)^18^	Retrospective, pediatric/adolescent	All developmentally & neurologically normal; excluded abs, myo sz, brain lesions, developmental delay	108	1 month–19 years	Mean 17.7 ± 7.8 months: 70	38	46 (42.6)	30/70	16/38	No ASM before recurrence	NI
Koutrou‐manidis^a^ (2018)^19^	Prospective, adults	Only GTCS 33 already with other minor seizure types	150	15–84 years (mean: 37.2 years; median: 31 years)	Mean 3.1 ± 2.1 years: 100	50	103 (68.7)	74/100	29/50	85 (57%)	<28 days
Shapiro (2019)^20^	Prospective, adults	(Extended 6 h EEG; in 5 EEG after sz recurrence)	36	>16 years (median: 35 years)	4 years: 11	25	7 (19.4)	3/11	4/25	15 (42%)	Median: 62 days (range 29–251 days)
Arican^a^ (2021)^21^	Prospective, pediatric/adolescent		55	1 month–18 years; (mean: 3.5 years)	3 years: 32	23	27 (49.1)	21/32	6/23	No ASM before EEG	NI
Linka^a^ (2023)^22^	Retrospective, adults	Full study included ASS, but ASS not included in this analysis	282	14–95 years (of the total group)	5 years: 127	155	81 (28.7)	44/127	37/155	(208) 73.9% in the main group	NI
Özdemir (2023)^23^	Retrospective, pediatric/adolescent	Excluded epileptic encephalopathy, abs, myo sz and infantile spasms, cerebral palsy, epileptogenic lesions	108	1–18 years; (mean 6 years ± 4.5)	Mean: 2.2 years (range 1.2–4): 43	65	68 (62.9)	31/43	37/65	NI	Mean: 31.2 h (range 1–96 h)
De Stefano^a^ (2023)^24^	Retrospective, adults	Routine EEG lab referrals only. After “first seizure event”: final diagnosis non‐epileptic events in 410 of 1010. Included 77 with previous similar events	1010	16–98 years (mean: 53 years)	≥2 years: 241	769	134 (13.3)	46/241	88/769	478/501 with new onset epilepsy	Median: 1 day (0–465 days)
López‐Maza^a^ (2024)^25^	Retrospective, adults	>55 years of age and epileptic seizure of unknown cause. Excluded epileptogenic lesions, established dementia, EEG features of GGE	87	59–94 years (mean 71.5 years ± 8.1)	Mean 7.3 ± 4.9 years: 49	38	38 (43.7)	19/49	19/38	65 (74.7%)	Median: 28 days (0–3733) 69 before seizure recurrence
Saleh (2024)^26^	Retrospective, pediatric/adolescent	Excluded <1 year follow‐up, those prescribed ASM, past CNS infection and head injury, abs, myo sz and infantile spasms, rolandic sz with centrotemporal spikes	317	1–18 years (median: 3 years)	Median 1.7 years (≥1 year): 305	12	260 (82.0)	254/305	6/12	No ASM before recurrence	NI
Joelsson (2025)^27^	Retrospective, adults	EEG lab referrals only Excluded intellectual disability, missing neurologist assessment, non‐attendance at EEG	252	17–88 years; (mean: 44 years)	Mean 4.4 years (range .33–6.93): 70	182	15 (5.9)	11/70	4/182	21 before EEG (8.3%)	Mean 61 days (range 0–358)

Abbreviations: abs, absence seizures; ASM, anti‐seizure medication;ASS, Acute symptomatic seizure; CNS, central nervous system; CT,computed tomography; GGE, genetic generalized epilepsy; GTCS, generalized tonic–clonic seizure; myo, myoclonia; *N*, number; NI, no information; pts, patients; SE, status epilepticus; sz, seizure.

^a^ Authors were contacted and generously provided complementary unpublished information.

### 2.2 Data definition, extraction

We used a comprehensive data extraction form to collect essential information from the selected studies. We included year of publication, study design (prospective vs retrospective), study population (distinguishing between children and adults), patient selection criteria, total number of participants, number of patients included in the analysis (if different from total number of participants), follow‐up duration after the first seizure, and where possible whether patients received antiseizure medications (ASMs), the time elapsed until the initial EEG was performed, the number of EEG channels used, and duration of EEG recordings. We included routine diagnostic EEG of any duration (e.g., routine (usually 20–30 min) or prolonged (often ≥1 h)), with or without sleep or other activation procedures.

To calculate the recurrence rate and derive sensitivity, specificity, and other statistical measures related to the diagnostic yield of the EEG, we extracted the number of patients with and without seizure recurrences. EEG findings were dichotomized as: (1) normal or with non‐specific EEG abnormalities (e.g., focal or diffuse slowing) and (2) demonstrating interictal epileptiform discharges (IEDs). Although we defined IEDs as interictal EEG patterns closely associated with epilepsy, such as spikes, spike‐waves, sharp waves, and sharp slow wave complexes, either with a focal or generalized distribution,^4^ no studies provided specific diagnostic or reporting criteria for IEDs so we relied on author reporting of the EEG findings. Although temporal intermittent monomorphic rhythmic delta activity (RDA) is considered an IED by most authorities, it was reported as an epileptiform abnormality in only one of the studies screened.^24^ No studies investigated the possible dose‐dependent relationship between the frequency of occurrence of IEDs and the risk of seizure recurrence.

Our focus was restricted solely to the reporting by authors of IEDs in EEG recordings. The association of seizure recurrence with other pathological EEG findings, such as focal or generalized slow activity, as well as more sophisticated EEG analyses like connectivity analysis, were deliberately excluded due to their limited clinical utilization and the considerably heterogeneity of published works on the latter.

Follow‐up durations were also determined based on the available heterogeneous data reported in the included studies (Table 1).

To ensure the reliability of our findings regarding the diagnostic yield of EEG after a first unprovoked seizure, studies were required to provide information on duration of follow‐up as well as sensitivity and specificity with confidence intervals for EEG findings, or absolute patient numbers that allowed us to calculate these statistics. For studies with unclear or unavailable data, efforts were made to contact the authors directly to obtain the necessary information as shown in Table 1.

### 2.3 Data analysis

We performed a random‐effects meta‐analysis of the pooled odds ratios (ORs) for seizure recurrence based on presence or absence or IEDs on the EEG. The available data limited the analysis of these time‐to‐event outcomes as binary. The *I* ^2^ statistic was used to assess the level of heterogeneity in the studies, with exploration of the sources of heterogeneity and sub‐group meta‐analyses as data allowed. In addition, we performed a meta‐analysis of diagnostic accuracy to determine the sensitivity, specificity, and receiver‐operating characteristic (ROC) curve of IEDs in the EEG to predict seizure recurrence, stratified by age of the population (children, adults). Publication bias was assessed visually with a funnel plot and using Egger's test. Meta regression was used for sensitivity analyses of the influence of variables such as patient age, study design, duration of follow‐up, year of publication, and estimated proportion of patients receiving ASM at some point. When these data were expressed as a range in individual studies, we used the mid‐point of this range as the point estimate for the analyses. Analyses were performed using the meta regression, metan, metadta, and midas modules in StataNow 19.

## 3 RESULTS

**FIGURE 2:**
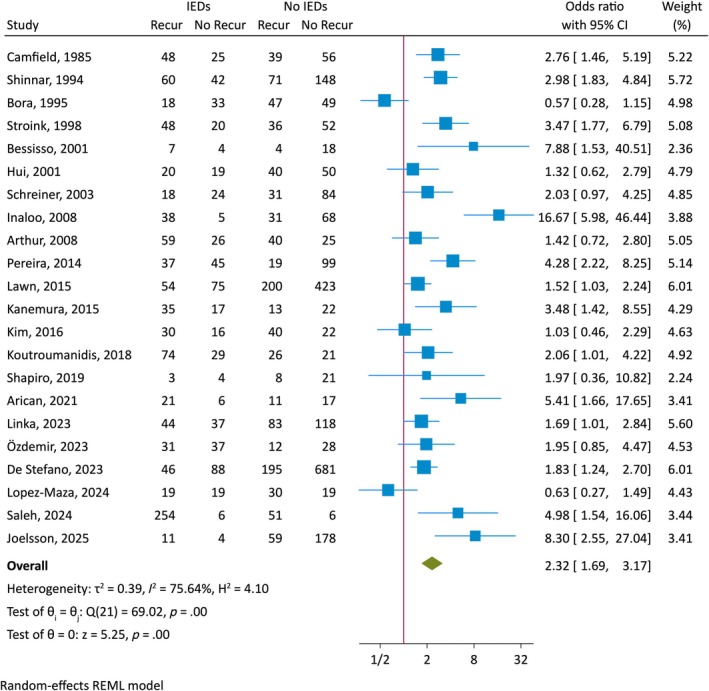
Forest plot of the association between interictal epileptiform discharges (IEDs) reported on the initial EEG after the first seizure and seizure recurrence. CI, confidence interval; Recur, recurrence.

Twenty‐two articles fulfilled the inclusion criteria, encompassing a total of 4847 patients (mean ± standard deviation [SD] patient number per study of 220 ± 232, median 150, range: 33–1010) and were included in the analyses (Table 1, Figure 2).^6^, ^7^, ^8^, ^9^, ^10^, ^11^, ^12^, ^13^, ^14^, ^15^, ^16^, ^17^, ^18^, ^19^, ^20^, ^21^, ^22^, ^23^, ^24^, ^25^, ^26^, ^27^ Thirteen of 22 studies^6^, ^8^, ^9^, ^10^, ^11^, ^13^, ^14^, ^15^, ^16^, ^17^, ^19^, ^20^, ^21^ were prospective. Ten studies included only adults^6^, ^9^, ^12^, ^13^, ^19^, ^20^, ^22^, ^24^, ^25^, ^27^ and 12 included only children.^7^, ^8^, ^10^, ^11^, ^14^, ^15^, ^16^, ^17^, ^18^, ^21^, ^23^, ^26^ Patient age ranges varied widely across individual studies, spanning 13–98 years in adult and 1 month to 19 years in pediatric/adolescent studies (see Table 1). Patient recruitment methods showed major differences, with EEG laboratory referrals,^7^, ^12^, ^24^, ^27^ hospital emergency department attendances, hospital admissions, outpatient attendances, or a combination of these. Only four studies specifically referred to recruitment by a first seizure clinic or service.^6^, ^18^, ^19^, ^24^ Patient selection criteria were also heterogeneous. Individual studies for various reasons excluded absence and myoclonic seizures, developmental and epileptic encephalopathies, focal seizures, or those with epileptogenic brain lesions, intellectual disability, or certain epilepsy syndromes (see Table 1). Follow‐up duration varied widely across studies, but was at least 2 years in most studies, with a range of 1–15.3 years (mean = 3.0 years, median = 2.5 years). The availability of data for the variables of interest differed across studies. The proportion of patients exposed to ASM was highly variable and delay to EEG was estimated in only 11 studies and showed prominent variability (Table 1).

### 3.1 Overall seizure recurrence

Overall, seizures recurred in 2061 of 4847 patients (42.5%) when combining all studies at any point during the variable follow‐up periods. The proportions with seizure recurrence across all studies ranged from 24% to 96%, with a random‐effects pooled estimate of 47% (95% confidence interval [CI] 40%–55%), 53% (95% CI 43%–64%) in children, and 40% (95% CI 32%–49%) in adults. However, these studies showed considerable heterogeneity with respect to time‐to‐event data and follow‐up durations.

### 3.2 IEDs reported on EEG and seizure recurrence

**FIGURE 3:**
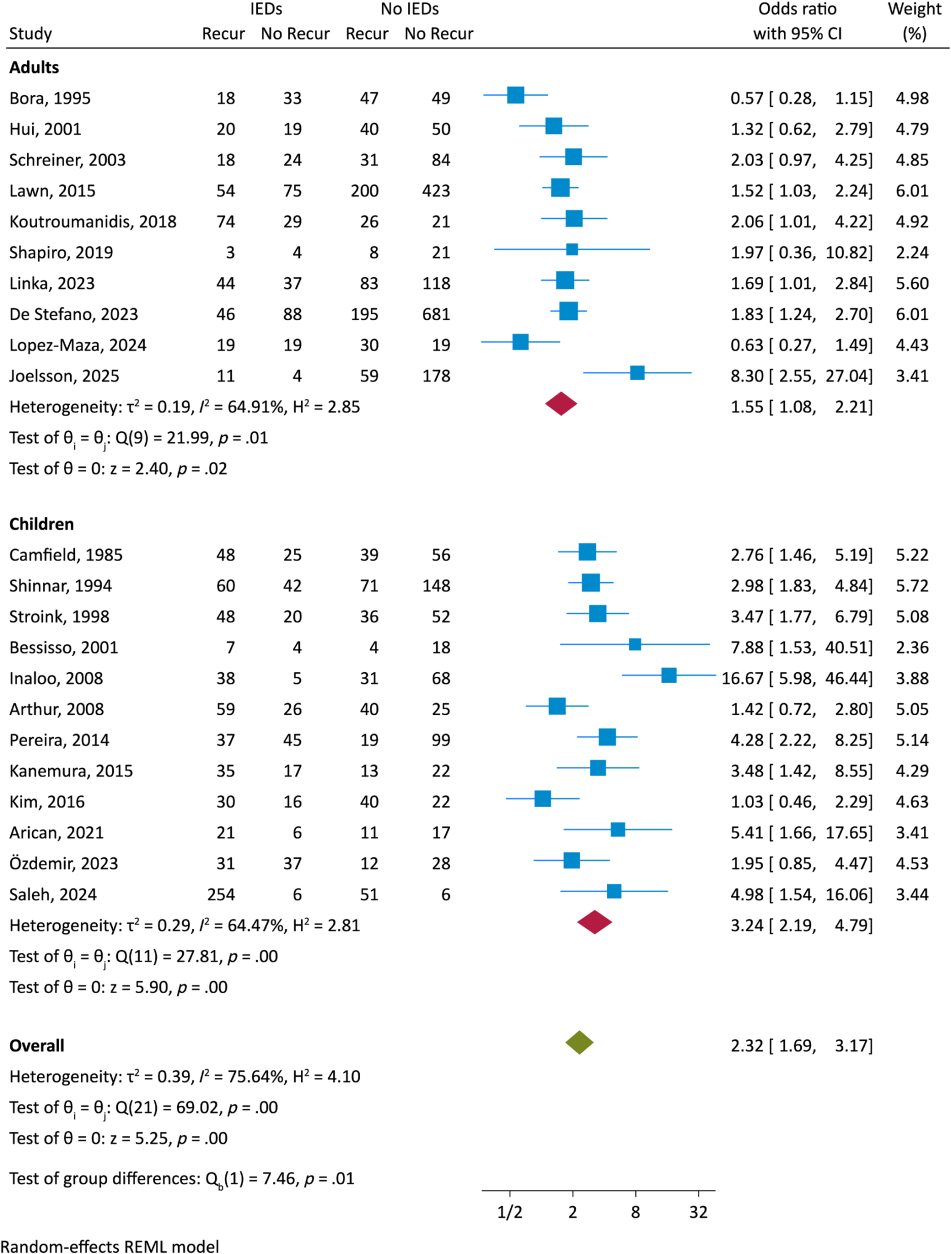
Forest plot of the association between interictal epileptiform discharges (IEDs) reported on the initial EEG after first seizure and seizure recurrence, grouping adults and children separately. CI, confidence interval; Recur, recurrence

Seizures recurred in 975 of 1556 patients with IEDs reported on the EEG (overall random‐effects proportion was 60% [95% CI 53%–68%]), and in 1086 of 3291 patients without IEDs on EEG (overall random‐effects proportion 40% [95% CI 33%–48%]), corresponding to a random‐effects pooled risk difference of 20% (95% CI 5%–35%). The overall meta‐analysis demonstrated an elevated OR for seizure recurrence in those with IEDs (OR 2.32, 95% CI 1.69–3.17, *p* < .001) (Figure 2), but with considerable heterogeneity (*I* ^2^ 76%, *p* < .001) and with some small‐study effects (Egger test *z* 2.1, *p* = .04) (Figure S2). Looking separately at adults and children, the risk of seizure recurrence when the EEG demonstrated IEDs remained significantly elevated in both age groups, but was higher in children, with an OR of 3.24 (95% CI 2.19–4.79) compared to adults 1.55 (95% CI 1.08–2.21), with prominent heterogeneity in both groups (Figure 3).

To further explore potentially important sources of the high heterogeneity, we meta‐regressed age (children vs adult), retrospective vs prospective design, duration of follow‐up, year of publication, and estimate of proportion exposed to ASMs on the predictive ability of IEDs for seizure recurrence. None had a significant effect on the likelihood of seizure recurrence (Figure S3 with log odds ratios).

In eight studies^10^, ^12^, ^13^, ^16^, ^17^, ^18^, ^21^, ^26^; (6 pediatric, *n* = 1209 patients, 923 children) patients did not receive ASMs before seizure recurrence. Of the remaining studies, a variable proportion of patients received ASMs prior to seizure recurrence, with an overall mean of 49% (range 8%–75%) of patients receiving treatment, with similar proportions in children and adults. Data on compliance with and durations of ASM treatment were unavailable. The overall random‐effects pooled proportion with seizure recurrence in untreated adults was 39% (95% CI 24%–54%) vs 41% (95% CI 30%–51%) in studies with initial treatment, and in untreated children was 59% (95% CI 41%–78%) vs 47% (95% CI 38%–56%) with initial treatment. If IEDs were present on EEG in untreated patients, the overall random‐effects pooled proportion with recurrence was 65% (95% CI 52%–79%) vs 45% (95% CI 29%–61%) if no IEDs were present—not significantly different from the studies with a proportion of treated patients (57% [95% CI 48%–66%] and 37% [95% CI 30%–44%], respectively). The random‐effects pooled data also showed no significant difference in the likelihood of recurrence related to IEDs when comparing those studies in which some patients were initially treated (OR 2.17, 95% CI 1.39–3.37) and those untreated (OR 2.66, 95% CI 1.74–4.06), including no difference in the studies of children or adults (Figure S4). In meta regression analyses, the effect of withholding ASMs was not significant (Figure S3).

We aimed to explore the relationship between the timing of EEG after the first seizure and the likelihood of detecting IEDs. However, 11 studies did not provide specific information about the delay in performing the EEG,^7^, ^8^, ^9^, ^10^, ^11^, ^14^, ^15^, ^18^, ^21^, ^22^, ^26^ and the remaining studies reported delays of <48 h,^13^ <96 h,^23^ <36 days,^12^, ^17^, ^19^ and 3 months^16^; medians of 1 day,^24^ 10 days,^6^ 28 days,^25^ and 62 days^20^; and a mean of 61 days^27^ (Table 1). This limited information did not show a significant correlation of the proportion of EEG recordings showing IEDs with the mean or maximal delay.

### 3.3 Sensitivity and specificity for seizure recurrence

The meta‐analysis of diagnostic accuracy revealed a random‐effects pooled sensitivity for IEDs of 48% (95% CI 39%–57%) and specificity of 72% (95% CI 64%–79%). The corresponding values were 62% (95% CI 54%–69%) and 67% (95% CI 58%–76%) in children, and 31% (95% CI 22%–43%) and 77% (95% CI 64%–87%) in adults (Figure S1). The random‐effects positive likelihood ratios for seizure recurrence were 1.91 (95% CI 1.48–2.46) for children and 1.39 for adults (95% CI 1.04–1.86), and negative likelihood ratios for seizure recurrence were .56 (95% CI .47–.68) for children and .89 for adults (95% CI .81–.96). Given the overall estimates of seizure recurrence of 53% in children and 40% in adults, the presence of IEDs would increase the overall probability of seizure recurrence to 68% (95% CI 63%–73%) in children and to 48% (95% CI 41%–55%) in adults, and their absence decrease it to 39% in children (95% CI 35–43) and 37% (95% CI 35–39) in adults.

In the studies of children that reported more specific EEG or clinical findings,^7^, ^8^, ^16^, ^17^ Rolandic discharges were usually the most common IED^8^, ^17^ or self‐limited epilepsy with centrotemporal spikes (SeLECTs) formed the majority of patients with IEDs and seizure recurrence.^8^, ^16^

**FIGURE 4:**
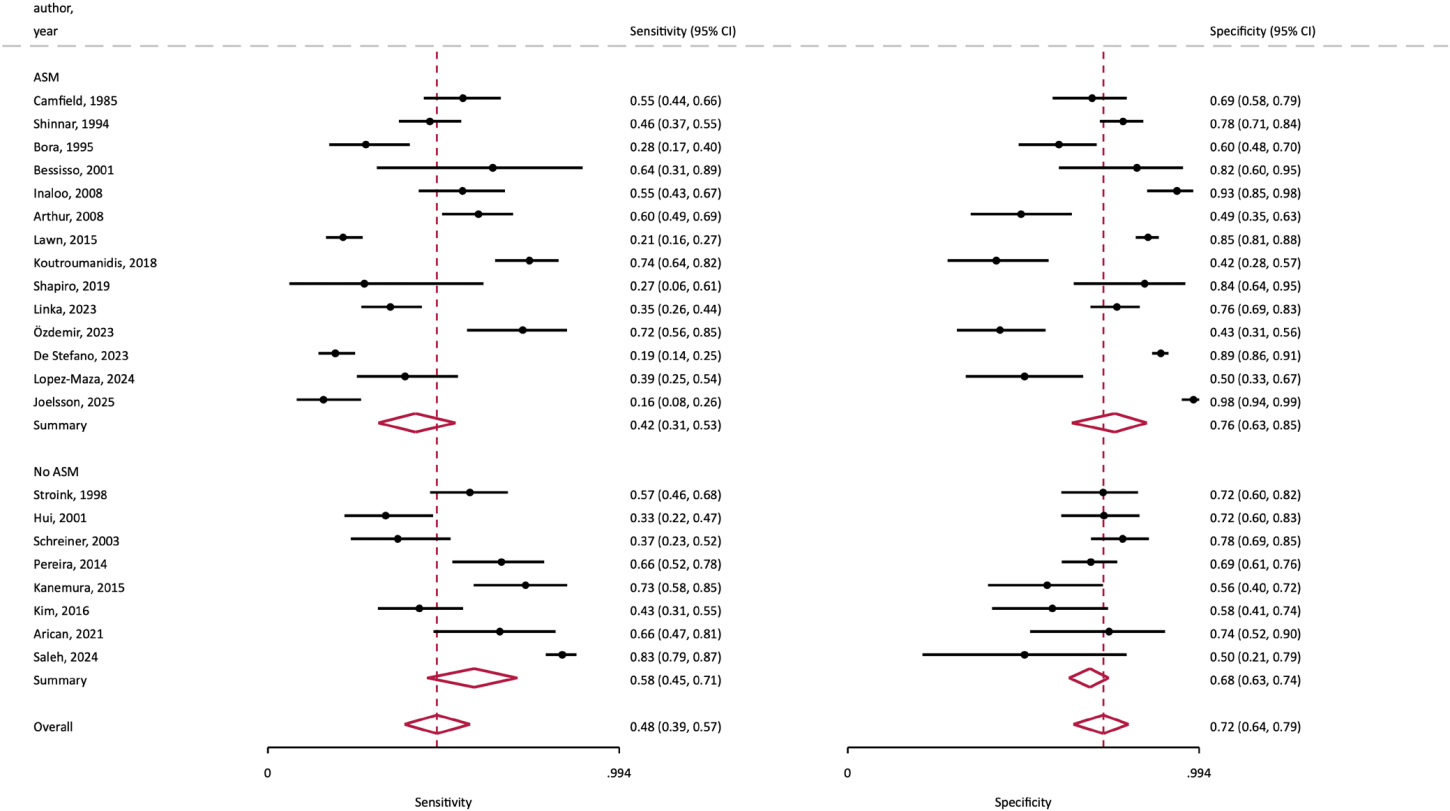
Forest plot of random‐effects sensitivity and specificity of interictal epileptiform discharges (IEDs) for seizure recurrence, grouping studies with antiseizure medication **(ASM)** and without a proportion of patients being treated with ASM **(No ASM)** after the first seizure.

In the eight studies^10^, ^12^, ^13^, ^16^, ^17^, ^18^, ^21^, ^26^ (*n* = 1209, 923 children) in which the patients remained untreated after the first seizure, the overall random‐effects pooled proportion with IEDs was 47% (95% CI 34%–59%) compared to 35% (95% CI 25%–45%) for those studies where some patients were treated with ASM, and were not significantly different either for children or adults. The presence of IEDs in untreated patients had a pooled sensitivity of 58% (95% CI 45%–71%) and specificity of 68% (95% CI 63%–74%) for seizure recurrence (Figure 4), no different from those studies in which some patients were treated with ASMs, where the pooled sensitivity and specificity were 42% (95% CI 31%–53%) and 76% (95% CI 63%–85%), respectively. The studies that included sufficient treatment information variously reported that ASM treatment either reduced the risk of seizure recurrence^19^, ^22^ or made no difference.^6^, ^7^, ^9^, ^11^, ^14^

## 4 DISCUSSION

Our primary objective in this meta‐analysis was to determine the added diagnostic value of EEG in predicting seizure recurrence after a first‐ever unprovoked seizure. Indeed, the presence of IEDs after the first unprovoked seizure was significantly associated with seizure recurrence in all age groups. Overall, 60% of patients with IEDs had a recurrence vs 40% without IEDs, with a post‐test probability of seizure recurrence of 60%. In children, the post‐test probability of seizure recurrence with the presence of IEDs was 68%, fulfilling the current ILAE criteria for the diagnosis of epilepsy after a first seizure, commonly benign childhood epilepsy with centrotemporal spikes, whereas in adults the post‐test probability of seizure recurrence was 48%.

In 2015, Krumholz et al. conducted a systematic review of relevant publications, following the American Academy of Neurology's classification of evidence criteria.^28^ They reported that in adults with an unprovoked first seizure, an EEG revealing IEDs showed an increased risk of seizure recurrence (Level A). Other factors, such as a prior brain insult (Level A), significant brain‐imaging abnormality (Level B), and nocturnal seizure (Level B) were also associated with an elevated recurrence risk.^28^

EEG together with brain imaging is used extensively as diagnostic tools in patients suspected of having epilepsy and after first seizure. Magnetic resonance imaging (MRI) detects potentially epileptogenic structural lesions in 14%–44% of patients with new‐onset epilepsy and, although valuable, MRI often cannot distinguish between non‐epileptogenic and relevant epileptogenic lesions.^29^, ^30^, ^31^, ^32^, ^33^ On the other hand, interictal EEG remains the most relevant test to enhance clinical diagnosis after a first seizure, including classification of new‐onset epilepsy.

Overall, the presence of IEDs on EEG significantly increases the risk of seizure relapse in our analyses. Most of the individual publications studied (Table 1) show an elevated risk of seizure recurrence. It is important to highlight that the duration of follow‐up in most included studies was much shorter than the 10‐year period specified in the ILAE definition, even if seizure recurrence reaches a plateau over time. In addition, seizure recurrence risk rapidly declines with time, falling rapidly with increasing duration of seizure freedom, and is below the 60% threshold within 3–12 weeks, independent of the underlying epilepsy syndrome.^6^ Many patients receive ASMs after their first unprovoked seizure, especially when paraclinical exams are positive, and this may be a confounding factor, albeit not evident in this current meta‐analysis (Figure 4).

### 4.1 The effect of age on seizure recurrence

Our data indicated that EEG recordings with IEDs are associated with higher seizure recurrence rates, especially in children. This finding is due mainly to the appearance of various epilepsy syndromes in this population, related to a genetic cause or predisposition, including SeLECTs, the most common epilepsy syndrome in children. In four of the five pediatric studies that reported more specific EEG or clinical findings, Rolandic discharges were usually the most common IED^8^, ^17^ or SeLECTs the most common epilepsy syndrome.^8^, ^16^ Children with SeLECTs frequently exhibit IEDs but have a low risk of relapse,^34^, ^35^ and treatment is not necessarily required. Therefore, it is crucial to relate clinical and EEG findings to specific epilepsy syndromes whenever possible, as this information holds great importance, especially in making management and treatment decisions in children and the potential influence of IEDs after a first seizure.

It should also be noted that healthy controls, both children and adults, may show epileptiform discharges. IEDs can be observed in healthy children without any history of seizures, with an overall prevalence up to 6.5%.^36^ Furthermore, a recent 24 h ambulatory EEG study from the United States on the basis of expert consensus found that 4.7% of healthy controls >50 years of age had IEDs.^37^ However, the interpretation of some EEG patterns still can differ in both children and adults.

A previous review has found the prevalence of IEDs on EEG in various prospective and retrospective studies combining first unprovoked seizures and new‐onset epilepsy showed a wide variation, ranging from ~18%–56% in children and 12%–50% in adults,^34^ similar to our findings with a range of 30.3%–82.0% in children and 6.0%–68.7% in adults.

### 4.2 Timing and duration of EEG recording after the first unprovoked seizure

The timing of EEG may be of crucial importance. Although no uniform standards for EEG timing and methodology following a first seizure currently exist, some studies show that EEG studies performed within 24 h after the seizure lead to higher IED detection, increasing from 34% to 51% in mixed populations of children and adults^33^ and from 19% to 28% in patients >16 years of age.^24^ It is of note that in EEG recordings of young children, the likelihood of recording IEDs is higher than in adults. Unfortunately, the studies eligible for our meta‐analysis did not systematically report the details of intervals between EEG recordings and the first seizure. One study that matched our inclusion criteria performed EEG within 48 h of a first seizure^13^ but did not report an advantage or significant association with IEDs and recurrence rates. However, this adult study excluded patients presenting with absence and myoclonic seizures.

The duration of EEG and use of activation procedures may also influence the diagnostic yield. Longer EEG duration enhances EEG sensitivity. In the current studies, EEG recording duration varied, with or without sleep recording and sleep deprivation (Table 1). Recording sleep also increases the yield of EEG.^24^ Koutroumanidis and Bruno suggested that sleep‐deprived EEG is preferable to early routine EEG for the diagnosis and classification of new‐onset epilepsy in an adult population.^19^ De Stefano suggested that the combined use of MRI and sleep‐EEG provides the highest diagnostic yield, both for the presence and absence of epilepsy.^24^ Due to the different EEG timings, methodologies, and lack of details in the articles included in our meta‐analysis, the optimal timing, EEG duration, and activation methods remain unclear.

### 4.3 Impact of treatment after the first unprovoked seizure

Previously a diagnosis of epilepsy required at least two unprovoked seizures occurring at least 24 h apart. However, with the adoption of the new definition of the ILAE criteria, the diagnosis of epilepsy requires only one unprovoked seizure along with evidence from an auxiliary examination of a comparable risk of recurrence to patients who have had two unprovoked seizure episodes.^2^ This shift in diagnostic criteria may have implications for evaluating the role of ASM treatment on seizure recurrence rate and in relation to detecting IEDs in the EEG.

However, this is a complex issue. Early treatment after the first seizure has not been found to alter long‐term prognosis and outcome, either in randomized controlled trials^38^ or in open‐label studies.^4^, ^6^, ^22^, ^39^, ^40^ However, a recent retrospective study has shown a significantly reduced likelihood of seizure recurrence up to 5 years after swift commencement of treatment within 48 h.^41^

The random‐effects pooled data in our meta‐analysis shows no significant difference in the likelihood of recurrence comparing studies in which some patients were initially treated vs those studies without initial treatment, and in meta‐regression analyses the effect of withholding ASMs was not significant (Figure 4, Figure S4). The studies in which a proportion of patients were treated (an overall mean of 49% [range 8%–75%] having ASM in our meta‐analysis) variously reported that ASM treatment reduced the risk of seizure recurrence^19^, ^22^ or made no difference.^6^, ^7^, ^11^, ^14^ Furthermore, Linka et al. found that the new definition of epilepsy was associated with an increased proportion of patients treated with ASM after first seizure but without a reduced recurrence rate.^22^ A major limitation and potential confounding factor is the highly variable follow‐up duration within and between the articles we have studied, and our need to analyze seizure recurrence as binary proportions without a specified follow‐up duration or time‐to‐event analyses. In addition, data on compliance with and durations of ASM treatment were unavailable.

With respect to the detection of IEDs, in the eight studies (six pediatric) where treatment was withheld until a second seizure occurred, no significant difference in the proportion of those with IEDs was evident when compared to studies where some patients were treated, neither in children^10^, ^16^, ^17^, ^18^, ^21^, ^26^ nor in adults.^12^, ^13^ Similarly, no significant difference was present in the pooled sensitivity and specificity of IEDs for recurrence when comparing these groups. However, these data have limitations, including the lack of information about whether EEG studies were conducted before or after commencing ASM treatment.

Eleven studies did not provide specific information about the delay in performing the EEG,^7^, ^8^, ^9^, ^10^, ^11^, ^14^, ^15^, ^18^, ^21^, ^22^, ^26^ whereas the remaining studies reported delays ranging from <48 h up until 3733 days. These relatively long and highly variable time intervals raise the possibility that patients may have already had seizure recurrence and received ASM treatment during this time. To gain a better understanding of the predictive value of IEDs, studies of patients while under full ASM treatment are warranted to determine if the increased recurrence rate is maintained.

Our meta‐analysis has found that the overall 72% specificity of IEDs for seizure recurrence was lower than the previously published studies of IEDs for epilepsy. This may be explained by differences in the study populations. We have analyzed seizure recurrence in studies where all patients have had or were initially thought to have had^24^ an unprovoked seizure, as compared to studies addressing the specificity of IEDs for patients with epilepsy and without epilepsy.

### 4.4 Limitations

Our meta‐analysis has multiple limitations. Substantial clinical and methodological heterogeneity exists among the included studies, including study design, patient recruitment methods, patient selection criteria and demographics, timing of EEG assessments, initiation of ASM in some patients before or after the initial EEG, and major variations in follow‐up durations.

In the absence of available survival data, we have used binary proportions to analyze seizure recurrence without a specified follow‐up duration, pooling patients with highly variable follow‐up durations within and between studies. This approach is not ideally suited for time‐to‐event outcomes, particularly because our pooled studies have highly variable and longer durations of follow‐up, considerable study heterogeneity, and high event probability. More patients without seizure recurrence tend to be lost to follow‐up, thus potentially overestimating seizure recurrence rate. Those with IEDs may also have longer follow‐up related to selection bias and therefore have a greater proportion with seizure recurrence. These limitations may influence both the overall ORs and diagnostic accuracy analyses.

We have also relied on author reporting of EEG abnormalities and have been unable to control for EEG recording techniques and EEG reporting accuracy and consistency. Agreement on what constitutes IEDs (focal vs generalized) and the many non‐epileptiform sharp transients and waveforms is needed for meaningful EEG interpretation. Most of the reviewed studies did not describe recording methods, including the number of electrodes, filter and sensitivity settings, and montages used.

In addition, we have pooled all patients together irrespective of seizure type, underlying etiology, and epilepsy syndrome, including both idiopathic and remote symptomatic first seizures. Similarly, all IEDs have been analyzed together, generalized and focal, and combining subgroups with different epilepsy syndromes such Rolandic IEDs and SeLECTS. Furthermore, we have not investigated the potential value of abnormal EEG recordings without IEDs in this study.

In the majority of the articles studied, information pertaining to whether ASM treatment was ongoing at the times of EEG and seizure recurrence was lacking.

Nonetheless, despite these limitations, we believe that we were able to extract the most critical parameters, enabling reasonable conclusions on the usefulness of EEG with IEDs after a first unprovoked seizure.

### 4.5 Future directions

In our meta‐analysis of IEDs on EEG after the first unprovoked seizure, several questions remain unsolved, including the influences of subgroup analyses: of idiopathic and remote symptomatic aetiologies and of electroclinical syndromes when definable; of focal and generalized seizures; of focal and generalized IEDs; of the frequency of occurrence of IEDs; and of the usefulness of other EEG abnormalities.

It is well known that routine diagnostic EEG studies in generalized epilepsy syndromes often show IEDs when recorded in the morning,^42^, ^43^ but their significance for relapse risk remains unknown. The number of electrodes used in EEG recordings is also important: the latest guidelines recommend the use of 25 electrodes to better cover the major lobes of both hemispheres, as well as anterior and basal temporal lobe structures.^44^, ^45^ This approach should be explored in future research.

It is also important to acknowledge that the quality and duration of EEG training varies markedly between countries and regions, leading to differences in the interpretation of what is considered pathological. Clinical misdiagnosis or incorrect reporting of the EEG can potentially do great harm, since it may be very difficult to reverse the diagnosis of epilepsy once made. Strategies to improve EEG reading and reporting quality are of utmost importance, and both the IFCN and ILAE are dedicating substantial resources to improve the competencies of neurophysiologists, neurologists, and neuropediatricians globally.

In a recent study of adults diagnosed with psychogenic non‐epileptic events, the EEG showed false‐positive IED patterns in 1.7%, and 0.5% of patients if neuroleptic medication effect is taken into account.^24^ In a study of the EEG recordings of 100 patients (54 with epilepsy and 46 with nonepileptic paroxysmal events), the most relevant aspects to define an IED correctly were identified by seven experts, providing an accuracy of 92%,^46^ and with similar accuracy of IED diagnosis in a study in children.^47^ However, in another recent large study, the interobserver agreement of nine experts in detecting IEDs in 1051 EEG recordings was considered only to be fair, which was attributed to the application of different thresholds by the experts.^48^ Efforts to improve the definition of IEDs and standardize what is considered to be an IED hold significant value, especially for individuals with less training and experience. Implementation of structured EEG reports, such as Standardized computer‐based organized reporting of EEG (SCORE), could also prove helpful, as well as incorporating the use of artificial intelligence support.^49^, ^50^

Epilepsy specialist care has been associated with incremental reductions in the hazard of premature mortality, and those referred to a comprehensive epilepsy program received the greatest benefits.^51^, ^52^ It should be kept in mind that clinical assessment is the gold standard for epileptic seizure and epilepsy diagnosis, assisted by relevant investigations such as EEG. Specialized first seizure clinics or services with standardized protocols and prospective data collection can optimize timely individual patient assessment and guide management, including further examining optimal EEG methodology and its timing. Treatment initiation should be based on an individual's examination by a competent health professional. It is important to note that the common misdiagnosis of epilepsy and the potential adverse effects of ASMs are crucial factors to consider when addressing a patient following a first unprovoked seizure.

In conclusion, our systematic review and meta‐analysis revealed that if the first EEG shows IEDs after a first unprovoked seizure, the risk of seizure relapse increases in both children and adults, and in children may surpass the threshold of 60% that is currently recommended for the diagnosis of epilepsy by the ILAE. EEG after a first seizure may influence further management depending on the epilepsy syndrome and risk–benefit analysis. However, the findings of this heterogeneous meta‐analysis should be interpreted with caution. It is paramount to avoid over‐reading of EEG/over‐interpretation of non‐epileptiform sharp transients. Most sharply contoured transients on EEG are normal variants or artifacts. Several areas require further investigation, specifically the optimal timing of EEG after the event, EEG duration, and activation strategies, such as awake vs sleep EEG recordings and subgroup analyses. These should be explored systematically to enhance the diagnostic accuracy and prognostic value of EEG. The effect of ASM on EEG patterns, and its correlation with seizure recurrence risk, also warrants further investigation for patients with a first seizure.

## AUTHOR CONTRIBUTIONS

Betül Baykan: study concept and design, acquisition of data, data analysis, first draft of manuscript, and review and editing of manuscript. John Dunne: study concept and design, acquisition of data, data analysis, review and editing of manuscript, and statistical analyses. Samuel Wiebe: study concept and design, acquisition of data, data analysis, review and editing of manuscript, and statistical analyses. Louis Maillard: study concept and design, acquisition of data, and review of manuscript. Sandor Beniczky: study concept and design, acquisition of data, data analysis, and review and editing of manuscript. Michalis Koutroumanidis: study concept and design, acquisition of data, and review and editing of manuscript. Margitta Seeck: study concept and design, study supervision, acquisition of data, data analysis, and review and editing of manuscript.

## CONFLICT OF INTEREST STATEMENT

B.B. has no conflict of interest to disclose relevant to this research activity. J.D. has received research funding from UCB Pharma and speaker honoraria from UCB Pharma and Eisai, but unrelated to this project. S.W. has received unrestricted educational grants on behalf of his institution from UCB Pharma, Paladin Labs, Jazz Pharma, and Eisai for work unrelated to this project and has served on advisory boards of Paladin Labs and Jazz Pharma. He has received speaker's fees from Torrent Pharma and Biopas Labs, for topics unrelated to this project. L.M. received consulting fees from Jazz Pharma, Angelini, UCB Pharma, and Bioserenity and was supported by the ANR (Agence Nationale pour la Recherche). S.B. has no conflict of interest to disclose relevant to this research activity. M.K. reports being a medical adviser to Piramidal Inc. M.S. reports shares in Clouds of Care and dEEGtal and has received speaker fees from Bial and Eisai. Margitta Seeck was supported by the Swiss National Science Foundation (No 180365). We confirm that we have read the Journal's position on issues involved in ethical publication and affirm that this report is consistent with those guidelines.

## Supporting information


Figures S1–S4.

## Data Availability

The data used for this meta‐analysis are available for research purposes upon reasonable request.

## References

[1] Amin U , Benbadis SR . The role of EEG in the erroneous diagnosis of epilepsy. J Clin Neurophysiol. 2019;36(4):294–297. 31274692 10.1097/WNP.0000000000000572

[2] Fisher RS , Acevedo C , Arzimanoglou A , Bogacz A , Cross JH , Elger CE , et al. ILAE official report: a practical clinical definition of epilepsy. Epilepsia. 2014;55(4):475–482. 24730690 10.1111/epi.12550

[3] Bouma HK , Labos C , Gore GC , Wolfson C , Keezer MR . The diagnostic accuracy of routine electroencephalography after a first unprovoked seizure. Eur J Neurol. 2016;23(3):455–463. 26073548 10.1111/ene.12739

[4] Hauser WA , Rich SS , Annegers JF , Anderson VE . Seizure recurrence after a 1st unprovoked seizure: an extended follow‐up. Neurology. 1990;40(8):1163–1170. 2381523 10.1212/wnl.40.8.1163

[5] Hauser WA . Commentary: ILAE definition of epilepsy. Epilepsia. 2014;55(4):488–490. 24731123 10.1111/epi.12587

[6] Lawn N , Chan J , Lee J , Dunne J . Is the first seizure epilepsy–and when? Epilepsia. 2015;56(9):1425–1431. 26222507 10.1111/epi.13093

[7] Camfield PR , Camfield CS , Dooley JM , Tibbles JA , Fung T , Garner B . Epilepsy after a first unprovoked seizure in childhood. Neurology. 1985;35(11):1657–1660. 4058756 10.1212/wnl.35.11.1657

[8] Shinnar S , Kang H , Berg AT , Goldensohn ES , Hauser WA , Moshé SL . EEG abnormalities in children with a first unprovoked seizure. Epilepsia. 1994;35(3):471–476. 8026390 10.1111/j.1528-1157.1994.tb02464.x

[9] Bora I , Seçkin B , Zarifoglu M , Turan F , Sadikoglu S , Ogul E . Risk of recurrence after first unprovoked tonic‐clonic seizure in adults. J Neurol. 1995;242(3):157–163. 7751859 10.1007/BF00936889

[10] Stroink H , Brouwer OF , Arts WF , Geerts AT , Peters AC , van Donselaar CA . The first unprovoked, untreated seizure in childhood: a hospital based study of the accuracy of the diagnosis, rate of recurrence, and long term outcome after recurrence. Dutch study of epilepsy in childhood. J Neurol Neurosurg Psychiatry. 1998;64(5):595–600. 9598673 10.1136/jnnp.64.5.595 PMC2170103

[11] Bessisso MS , El‐Said MF , Almula NA , Azzam SB , Sweid HA , Al‐Ali MG . Risk of seizure recurrences after first unprovoked seizure during childhood. Neurosciences (Riyadh). 2001;6(2):95–98. 24185269

[12] Hui AC , Tang A , Wong KS , Mok V , Kay R . Recurrence after a first untreated seizure in the Hong Kong Chinese population. Epilepsia. 2001;42(1):94–97. 11207791 10.1046/j.1528-1157.2001.99352.x

[13] Schreiner A , Pohlmann‐Eden B . Value of the early electroencephalogram after a first unprovoked seizure. Clin Electroencephalogr. 2003;34(3):140–144. 14521275 10.1177/155005940303400307

[14] Inaloo S , Sadeghi E , Rafiee M , Heydar ST . Risk of seizure recurrence following a first unprovoked seizure in childhood. Iran Red Crescent Med J. 2008;10(4):303–308.

[15] Arthur TM , deGrauw TJ , Johnson CS , Perkins SM , Kalnin A , Austin JK , et al. Seizure recurrence risk following a first seizure in neurologically normal children. Epilepsia. 2008;49(11):1950–1954. 19154398 10.1111/j.1528-1167.2008.01775.x

[16] Pereira C , Resende C , Fineza I , Robalo C . A 15‐year follow‐up of first unprovoked seizures: a prospective study of 200 children. Epileptic Disord. 2014;16(1):50–55. 24691297 10.1684/epd.2014.0643

[17] Kanemura H , Sano F , Ohyama T , Mizorogi S , Sugita K , Aihara M . EEG characteristics predict subsequent epilepsy in children with their first unprovoked seizure. Epilepsy Res. 2015;115:58–62. 26220377 10.1016/j.eplepsyres.2015.05.011

[18] Kim H , Oh A , de Grauw X , de Grauw TJ . Seizure recurrence in developmentally and neurologically normal children with a newly diagnosed unprovoked seizure. J Child Neurol. 2016;31(4):421–425. 26215392 10.1177/0883073815596616

[19] Koutroumanidis M , Bruno E . Epileptology of the first tonic‐clonic seizure in adults and prediction of seizure recurrence. Epileptic Disord. 2018;20(6):490–501. 30530414 10.1684/epd.2018.1014

[20] Shapiro M , Foster G . Prolonged EEGs in adult patients with a first unprovoked seizure: a prospective pilot study. Epileptic Disord. 2019;21(6):561–566. 31871008 10.1684/epd.2019.1110

[21] Arıcan P , Salman H , Dündar NO . Clinical profile and long‐term outcome of the first seizures in children. Turk J Pediatr. 2021;63(4):612–617. 34449143 10.24953/turkjped.2021.04.008

[22] Linka L , Magnus B , Habermehl L , Tsalouchidou PE , Zahnert F , Möeller L , et al. Effect of the revised definition of epilepsy on treatment decisions and seizure recurrence after a first epileptic seizure. Eur J Neurol. 2023;30(6):1557–1564. 36883241 10.1111/ene.15769

[23] Özdemir FMA , Öztoprak Ü , Atasoy E , Aksoy E , Çelik H , Ceylan N , et al. Characteristics and clinical value of early electroencephalography (EEG) after a first unprovoked seizure in children. Neurophysiol Clin. 2023;53(1):102848. 10.1016/j.neucli.2023.102848 36827816

[24] De Stefano P , Ménétré E , Stancu P , Mégevand P , Vargas MI , Kleinschmidt A , et al. Added value of advanced workup after the first seizure: a 7‐year cohort study. Epilepsia. 2023;64(12):3246–3256. 37699424 10.1111/epi.17771

[25] López‐Maza S , Abraira L , Bellido‐Castillo E , Lallana S , Campos‐Fernández D , Fonseca E , et al. Riesgo de epilepsia tras una primera crisis epiléptica de etiología desconocida en pacientes de edad avanzada [Risk of epilepsy after a first epileptic seizure with unknown etiology in elderly patients]. Rev Neurol. 2024;78(10):277–283. Spanish. 10.33588/rn.7810.2024055 38743021 PMC11407471

[26] Saleh DA , Hassan A . Seizure recurrence following the first unprovoked seizure: risk factors among children in UAE. Int J Dev Neurosci. 2024;84(8):905–917. 10.1002/jdn.10382 39364606

[27] Joelsson S , Andersson K , Brannefors P , Klemetz S , Gärdesmed L , Wennberg E , et al. Diagnostic value of EEG after a first unprovoked seizure in adults – a population‐based study. Epilepsy Behav. 2025;162:110151. 10.1016/j.yebeh.2024.110151 39615259

[28] Krumholz A , Wiebe S , Gronseth GS , Gloss DS , Sanchez AM , Kabir AA , et al. Evidence‐based guideline: management of an unprovoked first seizure in adults: report of the guideline development subcommittee of the American Academy of Neurology and the American Epilepsy Society. Neurology. 2015;84(16):1705–1713. 25901057 10.1212/WNL.0000000000001487 PMC4409581

[29] Hakami T , McIntosh A , Todaro M , Lui E , Yerra R , Tan KM , et al. MRI‐identified pathology in adults with new‐onset seizures. Neurology. 2013;81(10):920–927. 23925763 10.1212/WNL.0b013e3182a35193

[30] Fisch L , Lascano AM , Vernaz Hegi N , Girardin F , Kapina V , Heydrich L , et al. Early specialized care after a first unprovoked epileptic seizure. J Neurol. 2016;263(12):2386–2394. 27604619 10.1007/s00415-016-8272-3

[31] Ho K , Lawn N , Bynevelt M , Lee J , Dunne J . Neuroimaging of first‐ever seizure: contribution of MRI if CT is normal. Neurol Clin Pract. 2013;3(5):398–403. 29473604 10.1212/CPJ.0b013e3182a78f25 PMC5765827

[32] Tranvinh E , Lanzman B , Provenzale J , Wintermark M . Imaging evaluation of the adult presenting with new‐onset seizure. AJR Am J Roentgenol. 2019;212(1):15–25. 30299997 10.2214/AJR.18.20202

[33] King MA , Newton MR , Jackson GD , Fitt GJ , Mitchell LA , Silvapulle MJ , et al. Epileptology of the first‐seizure presentation: a clinical, electroencephalographic, and magnetic resonance imaging study of 300 consecutive patients. Lancet. 1998;352(9133):1007–1011. 9759742 10.1016/S0140-6736(98)03543-0

[34] Wirrell EC . Prognostic significance of interictal epileptiform discharges in newly diagnosed seizure disorders. J Clin Neurophysiol. 2010;27(4):239–248. 20634717 10.1097/WNP.0b013e3181ea4288

[35] Bergey GK . Management of a first seizure. Continuum (Minneap Minn). 2016;22:38–50. 26844729 10.1212/CON.0000000000000271

[36] Borusiak P , Zilbauer M , Jenke AC . Prevalence of epileptiform discharges in healthy children–new data from a prospective study using digital EEG. Epilepsia. 2010;51(7):1185–1188. 20002145 10.1111/j.1528-1167.2009.02411.x

[37] Lam AD , Sarkis RA , Pellerin KR , Jing J , Dworetzky BA , Hoch DB , et al. Association of epileptiform abnormalities and seizures in Alzheimer disease. Neurology. 2020;95(16):e2259–e2270. 32764101 10.1212/WNL.0000000000010612 PMC7713786

[38] Marson A , Jacoby A , Johnson A , Kim L , Gamble C , Chadwick D . Immediate versus deferred antiepileptic drug treatment for early epilepsy and single seizures: a randomised controlled trial. Lancet. 2005;365(9476):2007–2013. 15950714 10.1016/S0140-6736(05)66694-9

[39] Sharma S , Chen Z , Rychkova M , Dunne J , Lee J , Lawn N , et al. Short‐ and long‐term outcomes of immediate and delayed treatment in epilepsy diagnosed after one or multiple seizures. Epilepsy Behav. 2021;117:107880. 33711683 10.1016/j.yebeh.2021.107880

[40] Ren T , Li Y , Burgess M , Sharma S , Rychkova M , Dunne JW , et al. Long‐term physical and psychiatric morbidities and mortality of untreated, deferred and immediately treated epilepsy. Epilepsia. 2024;65:148–164. 38014587 10.1111/epi.17819

[41] Ménétré E , De Stefano P , Megevand P , Sarasin FP , Vargas MI , Kleinschmidt A , et al. Antiseizure medication ≤48 hours portends better prognosis in new‐onset epilepsy. Eur J Neurol. 2024;31(2):e16107. 37889889 10.1111/ene.16107 PMC11236038

[42] Fittipaldi F , Currà A , Fusco L , Ruggieri S , Manfredi M . EEG discharges on awakening: a marker of idiopathic generalized epilepsy. Neurology. 2001;56(1):123–126. 11148252 10.1212/wnl.56.1.123

[43] Labate A , Ambrosio R , Gambardella A , Sturniolo M , Pucci F , Quattrone A . Usefulness of a morning routine EEG recording in patients with juvenile myoclonic epilepsy. Epilepsy Res. 2007;77(1):17–21. 10.1016/j.eplepsyres.2007.07.010 17851038

[44] Seeck M , Koessler L , Bast T , Leijten F , Michel C , Baumgartner C , et al. The standardized EEG electrode array of the IFCN. Clin Neurophysiol. 2017;128(10):2070–2077. 28778476 10.1016/j.clinph.2017.06.254

[45] Rosenzweig I , Fogarasi A , Johnsen B , Alving J , Fabricius ME , Scherg M , et al. Beyond the double banana: improved recognition of temporal lobe seizures in long‐term EEG. J Clin Neurophysiol. 2014;31(1):1–9. 24492440 10.1097/WNP.0000000000000019

[46] Kural MA , Duez L , Sejer Hansen V , Larsson PG , Rampp S , Schulz R , et al. Criteria for defining interictal epileptiform discharges in EEG: a clinical validation study. Neurology. 2020;94(20):e2139–e2147. 32321764 10.1212/WNL.0000000000009439 PMC7526669

[47] Stroink H , Schimsheimer RJ , de Weerd AW , Geerts AT , Arts WF , Peeters EA , et al. Interobserver reliability of visual interpretation of electroencephalograms in children with newly diagnosed seizures. Dev Med Child Neurol. 2006;48(5):374–377. 16608546 10.1017/S0012162206000806

[48] Jing J , Herlopian A , Karakis I , Ng M , Halford JJ , Lam A , et al. Interrater reliability of experts in identifying interictal epileptiform discharges in electroencephalograms. JAMA Neurol. 2020;77(1):49–57. 31633742 10.1001/jamaneurol.2019.3531 PMC6806666

[49] Beniczky S , Aurlien H , Brøgger JC , Hirsch LJ , Schomer DL , Trinka E , et al. Standardized computer‐based organized reporting of EEG: SCORE – second version. Clin Neurophysiol. 2017;128(11):2334–2346. 28838815 10.1016/j.clinph.2017.07.418

[50] Tveit J , Aurlien H , Plis S , Calhoun VD , Tatum WO , Schomer DL , et al. Automated interpretation of clinical electroencephalograms using artificial intelligence. JAMA Neurol. 2023;80:e231645. 10.1001/jamaneurol.2023.1645 PMC10282956 37338864

[51] Lowerison MW , Josephson CB , Jetté N , Sajobi TT , Patten S , Williamson T , et al. Association of levels of specialized care with risk of premature mortality in patients with epilepsy. JAMA Neurol. 2019;76(11):1352–1358. 31380987 10.1001/jamaneurol.2019.2268 PMC6686748

[52] Hargreaves DS , Arora S , Viveiro C , Hale DR , Ward JL , Sherlaw‐Johnson C , et al. Association of quality of paediatric epilepsy care with mortality and unplanned hospital admissions among children and young people with epilepsy in England: a national longitudinal data linkage study. Lancet Child Adolesc Health. 2019;3(9):627–635. 31281027 10.1016/S2352-4642(19)30201-9

